# American Academy of Dental Science

**Published:** 1901-04

**Authors:** 

**Affiliations:** American Academy of Dental Science


### Reports of Society Meetings.

### AMERICAN ACADEMY OF DENTAL SCIENCE.

The regular monthly meeting of the American Academy of Dental Science was held at Young's Hotel, Boston, Wednesday evening, January 2, 1901, at six o'clock.

Two papers were read by Dr. D. D. Smith, of Philadelphia, Pa., the first entitled "Prophylaxis in Dentistry," the second entitled "A Unique Method of tightening or inserting Lower Front Teeth."

[Dr. Smith failed to furnish his first paper for publication. The substance, however, will be found in a paper by him published in Volume XXI., page 805.—Ed.]

*President Pond.*—We have to-night a subject of great interest to us. While every subject that we discuss here has for its object the advancement of our profession, and is therefore of great interest, some subjects are so practical, so perfectly adaptable to our every-day practice, that they seem of especial importance. The subject to-night is of very practical importance, a method of treatment that you can use every day.

DISCUSSION.

*Dr. Smith.*—Having read this paper, gentlemen, it has impressed me that I have not, after all, dwelt upon the actual methods to be pursued as minutely as I should have done. They are so clear in my own mind that perhaps I have omitted little matters of detail and not made it clear to you. I give you the privilege of asking any questions in relation to the paper and the subject I have dealt with, and I will endeavor to answer them to the best of my ability. But permit me to ask you a question. How many of you have practised at any time this method of oral prophylaxis? All that have, please hold up the hand.

*Voices.*—What method? What method?

*Dr. Hopkins.*—I do not think we quite understand the details of the method.

*Dr. Smith.*—Let me try to explain this method of prophylaxis in detail. In the first place, it is not all cleaning teeth. It does clean the teeth, but that is not all that is accomplished. If you will take one of these orange-wood sticks—there is a toughness in the orange-wood which makes it desirable—and some pulverized pumice-stone, and go over the surfaces of the teeth, using a well-sharpened stick and going well up into the festooning of the gum, even on what may appear to be a clean tooth, you will find that there is a greater difference in the appearance and in the "feel" after the operation than you would have predicted. The sense of touch will enable you to recognize that you have taken something off the tooth, although you may not be able to see what it is. Now, that condition exists on all surfaces of the teeth, not on the outside alone, but on the inside, between them, and even on the occlusal surfaces. The lower sixth- and twelfth-year molars and the wisdom-teeth on the lower jaw should be just as thoroughly and carefully polished on the inner or lingual faces as they are upon the outside or buccal; and all in between the teeth just so far as you can get your stick, reduced to a thin, wedge-shaped point, and pumice to go. Every tooth is to be thoroughly polished, all exudations from the gum, gatherings of food particles, the deposit from the saliva and mucus during the night are to be thoroughly removed, and every portion of the exposed surface of the tooth is to be left in a perfectly polished and smooth condition. That is what I mean by this treatment, gentlemen. I claim in the paper that it is utterly impossible to do this with the wheels. It must be done by hand and with hand instruments.

The treatment as outlined stimulates the teeth to throw out and deposit new and better tooth-material; the teeth under this stimulation become better; the internal vital functions seem roused to new activities. Give a patient three months, if you please, under this treatment, and after three polishings, whether you start with young teeth, teeth in middle life, or whether the subject be an elderly person, there is not an individual practitioner who will say that it has not benefited the teeth, or that the character of the tooth-material is not actually better than before you commenced the treatment.

*Dr. Andrews.*—Any massage at all with the fingers?

*Dr. Smith.*—Not at all. It is all done with sticks and pumice.

*Dr. Eames.*—Do you use the finest?

*Dr. Smith.*—Not the finest. Why pumice-stone? Because of the grit, and because the pumice-stone will float in the liquids of the mouth and there is no danger of its remaining between the gums and the teeth. I used to teach patients that it would not do to use pumice-stone because it would work down under the gums and cause their recession from the teeth. Instead of this, the pumice-stone floats and is immediately washed away.

*Dr. Boardman.*—Will you please pass around your instrument?

*Dr. Smith.*—Certainly. Considerable of the surface can be reached with just the plain stick, as you know. Some are larger than this one which you see here. Larger ones can be had, and they are better to handle. They can be kept in condition while working with them simply by whittling them down a little, and cutting off the brush end from time to time. I have half a dozen of the porte-polishers at hand, that I may not be inconvenienced and retarded when at work. And I have these two forms, but varying sizes of the sticks, and that is about all. But as I go on with the polishing I try to keep them in condition so they will do the work, not letting them get too soft at the points.

I wish to say, in regard to this instrument, that it was devised by Dr. Louis Jack years ago, not for this purpose, but for cleaning teeth after the old methods. It was for use after the occasional removal of tartar. Very few of these devices were ever sold, and they have so long been obsolete that the younger men know nothing of them. As an illustration of how they were appreciated by the profession, I had one in my office, and when I wanted some more, I was informed by the S. S. White people that they had none. They would not make a single one, but they would make them up for four dollars apiece if I would take half a dozen of them. I took the instrument to another maker, had it modified a little, and now they can be obtained in any quantity at a reasonable price from Dr. Ivory. It is precisely the same pattern of instrument, but a little smaller in some of its parts and more convenient for use in the mouth.

*Dr. Eames.*—What is the price of this?

*Dr. Smith.*—They retail, I think, for two dollars and twenty-five cents or two dollars and fifty cents. With these instruments, as can readily be seen as they are passed to you, any part of the mouth and any part of a given tooth can be easily reached.

A gentleman said to me the other day, "I have done something in the line of prophylaxis myself, doctor, and I think I know something about it;" and went at one of my patients, who was kind enough to submit to be presented to him, with a seeming determination to find some cavity or some fault in the mouth, I having said there had been practically no decay and but one filling placed in the teeth since the treatment commenced.

The prophylaxis which this gentleman a number of years ago had had something to do with was the suggestion of hot air for the treatment of devitalized teeth and for sensitive dentine; and he had also used it injected between the teeth to destroy the injurious agents which were at work there. Now, that is one thing, but it is not the prophylactic treatment which I am suggesting. Mine is a method of obtaining change of environment for the teeth in the mouth, a positive, systematic, and effectual change of tooth environment. It is by this method alone that I am able to accomplish it.

Another thing which has impressed me more recently is the peculiarly healthy condition which the gums take on after two or three treatments. That there is an exudation from the gums and from the alveolar tissue which lodges on the teeth is unmistakable. In this respect the teeth are analogous to the fingernails: leave them without due care, and there will be the same irritated condition, accompanied, in extreme cases, with pus discharge such as is often seen about the teeth.

A question from one of the gentlemen examining the cases in my office may well be answered here: "Do you go up into the festooning of the gums with your polishing instruments?" That, gentlemen, is one of the things that I do, go with the polishing instruments up to the festooning of the gums, and if I make them bleed I do not regret it at all. I do not aim to do that, but I do aim to remove from that part of the tooth, the cervical portion, all the exudation from the gums as well as all the extraneous matter there. In some cases it should be done oftener than once a month. But done once a month, it will produce stimulation to the gum-tissue, stimulation to the alveolar tissue, stimulation to the whole oral structures, inducing the most attractive and apparently the most perfect condition of the mouth attainable.

*Dr. Andrews.*—Would you use considerable force?

*Dr. Smith.*—Use whatever force may be necessary to get the surface absolutely polished or clean. You can tell very readily, after polishing a few sets of teeth in this way, when that is accomplished. Now, I ask you once more, How many have undertaken it?

*Dr. Meriam.*—I think we have all done something of it.

*Dr. Smith.*—I have no doubt you have cleaned teeth.

*Dr. Meriam.*—Cleaned for a purpose.

*Dr. Smith.*—Have you produced this condition of the mouth which I describe as the result of this system of treatment? If you have not, I want to ask of you, not for my benefit, but for your own, that you will undertake at least one case from this time for three months. Select a member of your own family, your wife, if you please, or one of your children who needs the treatment, and do it thoroughly and conscientiously. Do it in the manner we have endeavored to teach that it should be done. Discard power polishers, using only orange-wood sticks and pumice. At the end of three months note the results, and write to me giving your experience, and see if you are not as much astonished and gratified as I have been.

Do not attempt to bring it into all your practice. I have not done it in a day. It has taken three years to bring fifty patients fully under the system. I have not suggested it to every one. I have not felt secure and on a solid foundation until more recently, but I have done it now in a sufficient number of cases and with sufficient fidelity to know what the results are. If you have a breath in the family that is not always sweet, try it there, and see what the result will be on the breath.

I wish to make another statement to you. I will not place it in the paper, but I make this statement: that of the fifty patients, there is not one who has been six months under the treatment that has not improved in general health, some more than others.

*Dr. Andrews.*—About how much time is required for a treatment of this kind?

*Dr. Smith.*—It will depend upon the number of teeth in the mouth and the frequency with which you practise it. After becoming expert at it you will do it much more readily, and you will find that you will go over a set of teeth much easier after they have been treated two or three times. It requires from half an hour to an hour for a treatment.

*Dr. Eames.*—I would like to ask if you consider cases of loosening, so-called pyorrhœa, and in what stages of advancement, and with what success?

*Dr. Smith.*—Would you like to hear the results from one of the worst cases of pyorrhœa I have ever had for treatment? I invited ten gentlemen to my office, as I said, only last week for the purpose of seeing this case. The mouth had been under my care for at least fifteen years, but I had made no progress at all in the treatment of the case. It had rather been going backward all the time. It is one of those cases where the crowns are very large, the teeth constricted at the necks with short roots; a small mouth, exceedingly difficult to work in. During the years I have had the case in charge there has been a gradual increase in loosening, in recession of the gums, and in pus discharge. Two or three of the lower front teeth and some of the molars have been lost and the trouble seemed uncontrollable.

About a year ago I became convinced that this method of treatment would certainly do the most that could be done for the cure of pyorrhœa, and so I commenced vigorously with this patient. In the beginning I saw her once a month. At the end of the first month there seemed little change, but after three treatments I saw an improvement. And last June—she is a lady who lives in New York and had to come over to Philadelphia every month—she came to the shore to stay for the summer. I said, "You must come to my office once in two weeks." The teeth, those that were left, were quite loose. I had a little piece of bridge-work in the mouth, three teeth on two roots that had gotten loose. I thought all the teeth were going, and I felt very badly about it, because the patient had been so anxious to save her teeth. She came during the summer as desired, once in two weeks. And, gentlemen, I have to say to you that to-day those gums have taken on as healthy a condition as could possibly be desired. The alveolar process has not returned; the gums have not closed down where they originally were, but they are in a healthy condition. There is no discharge of pus, and the breath has lost the fetor which formerly pertained to it. And the teeth have tightened, until to-day they are perfectly comfortable. They are not absolutely tight, but they are so near it, and in such a healthy condition, that she masticates with perfect comfort.

I showed the case to at least eight gentlemen, some of the best practitioners of Philadelphia, as an example of what this treatment will do for pyorrhœa, it being one of the worst cases which I have ever had to treat.

What is the explanation of it? To me it is this: there is an exudation from the gums, or a discharge of pus from the alveolar structure and gums, which lodges at the cervix and at points which it is impossible for the patient to reach. Becoming inspissated at the constricted parts of the teeth, it undergoes decomposition, especially at night when the muscular system is relaxed and the mouth generally open. This toxic matter cemented on the teeth becomes more and more irritating. It is often attached to the roots of the teeth with great force. And how shall it be taken off? You know how Dr. Riggs used to do. It has to be removed by skilful manipulation. And having removed these irritating substances once, are we to abandon them and let the exudations from the unhealthy alveolar and gum structure reaccumulate? They begin at once to re-cement themselves on the teeth for their destructive work. What shall we do. Keep them off. Is not that the treatment? And if kept off, the pus discharge will stop and the gums will assume a healthy condition again. It is not medication that is required for pyorrhœa; it is rather relief from the irritation of toxic accumulations on and about the teeth. Frequent change of environment, with soothing local medication, is what is needed and what will certainly give the best results.

*Dr. Hopkins.*—Mr. President, I do not know how the other gentlemen have been affected by this paper, but I can say that it has been a great gratification to me to hear such enthusiasm, and that I am a firm believer in what the essayist has said. I believe that it is possible for a dentist to take a child through life without that child ever having a toothache, a dead tooth, or a serious operation, except in most extraordinary conditions. I think that we have all somewhat backslidden, and that such a paper as that which has just been given us is what we need to stimulate us to renewed effort.

I was brought up in the office of Dr. E. J. Dunning, of New York, and his successor, and I have always used the orange-wood stick in the manner described by the essayist to cleanse teeth.

It is, I think, a perfectly safe thing for a dentist to say to a parent, if you will leave the child in my hands and allow me to see the child when I send for him, I can promise you security from any serious trouble.

It seems to me that we need to set our standard very high, so high that we will have to keep struggling all the time to keep up to it. I do not think it is as difficult a matter to control our patients as some seem to feel. I think that it is just as well to control our practice as to have our patients control us. It is much more satisfactory.

It is my practice now, with a great many of my patients, when they leave the office, not to say to them, come in again in three months, or in six months, but to say, I will send for you when I want you, and I expect you to respond. If I find there is nothing to be done, the patient is usually grateful, and is willing to pay the fee for examination. If I find that there is an accumulation of work, that large cavities exist, I am mortified because I have made an error in judgment. I ought to have sent for the patient earlier. I never have carried my practice to the extreme that the essayist has suggested, but I have been weak, I realize, weak in not asserting what I have believed to be the truth. I consider this an extremely helpful and timely paper.

*Dr. Smith.*—Allow me to interrupt you just here, as the gentleman made a remark which reminds me of one thing I had forgotten, and that is in regard to the way I deal with these fifty patients that I speak of. I give them at the time of treatment an appointment for the following month. I have my appointment book so arranged that A coming to-day for this treatment takes an appointment for the same day the next month. When I speak of having fifty cases, I mean fifty cases where I have a regular standing appointment with them from month to month.

*Dr. E. H. Smith.*—Permit me to ask the essayist if he uses polishing-strips between the teeth, or is he able to reach all the inaccessible places with the orange-wood sticks properly shaped?

*Dr. D. D. Smith.*—In reply to that I wish to say that I never use the strips at all as part of this treatment, but I do frequently use silk for going in between the teeth. I rely on the sticks properly shaped to get in between the teeth. Understand me, I do not expect to stop every particle of decay. My belief is that nature placed teeth close together, in the position that we find them, for a purpose. I certainly should not feel that it is necessary, and it has not proved necessary, to separate them for the purpose of getting strips, or anything of that nature, between them for polishing. It can be done altogether with these sticks, supplemented where necessary with floss silk.

Another thing I wish to say, that I enjoin the greatest care on the part of patients in the use of the brush; they are expected to have what I conceive to be a good brush, and I insist upon their using it, and also a good tooth-powder. There is what I consider a good tooth-powder made here in New England,—Caulder's Dentine. And then I request patients to use fine salt at least once a week. Other than that I do not exact anything from them in the way of cleaning. But if they come to me with the teeth showing neglect, they always get scolded, and particularly the children and the youth that are under the treatment. I am very careful to enjoin upon them that they must exercise care as well as myself.

*Dr. Brackett.*—In the paper which has been put before us this evening there are many points about which I would like to speak; but if I said all that has come to my mind, there would be no opportunity for any one else. I think that we, as members of the Academy, should be able to disabuse the mind of the essayist of the idea, if it is there, that Dr. Taylor has been other than an earnest admirer of his practice and an honest representer of its merits. So far as my acquaintance goes, Dr. Taylor has done more than any other dentist in this region to put in practice Dr. Smith's methods. To this he gave testimony when he appeared before us last May; and with an increasing number of patients and with gratifying results, he has continued the practice up to the present time. Those who heard Dr. Taylor's paper and remarks will testify that he gave great credit to Dr. Smith, and that he did not claim for himself originality or any personal glory whatever.

I was a student in the office of Dr. Taylor. For years we both had the privilege and advantage of an intimate acquaintance and intercourse with Dr. Riggs; and it is true, as our essayist has well said, that Dr. Riggs is deserving of honor. I believe we all appreciate that he was a pioneer in pointing out the local etiology of pyorrhœa alveolaris, and in thoroughly and successfully treating it with peculiarly shaped instruments of his own devising and of far-reaching capacity. With them he sought to remove from the teeth, with the utmost particularity, all calcareous deposits, however far beneath the gum they had penetrated. He taught also the removal of the necrotic border of the alveolar process, usually present in deep-seated cases, so that there should be left only clean vital tissues. And he followed the use of the scalers with an earnest use of the stick and pumice-stone. Unless in the case of a few of his disciples, I have never seen in the hands of any other practitioner such use of the stick as I have seen in Dr. Riggs's hands. It was his practice and his inculcation in his teaching that not only should the scaler remove the calcareous deposits, however far beneath the gum they might extend, but that the orange-wood stick, cut to a long, thin taper, should follow till all that portion of the root to which foreign material had been attached, as well as the crown, was made thoroughly clean and polished.

No one would claim for any practitioner who lived during the period which Dr. Riggs's lifetime covered that he did in all things what we are able to do to-day. I do not think that any of those who were most familiar with Dr. Riggs's practice would claim that he ever instituted any such thing as the systematic and thorough cleaning and polishing of the teeth once a month.

*Dr. Smith.*—For the purpose of arresting decay?

*Dr. Brackett.*—For any purpose whatever. It was Dr. Riggs's practice to go over a case of pyorrhœa as thoroughly as he could in the first operation, or series of operations. Then he instructed the patient to return after an interval of a week or two, in order that he might have an opportunity to revise the treatment, if revision were needed. Wherever he could see that the former operation had failed to accomplish all that it should, as manifest in the tone and color of the gum, in suppuration, or in any of the ways which are familiar to us, there he wished to make a revision, and to make complete that which the first operation had left incomplete. Following all, Dr. Riggs taught his patients the great value of their own efforts in maintaining cleanliness of the teeth, and the importance of their having any new accumulations of calculus removed at intervals so short that it should not have an opportunity to penetrate again beneath the gum.

*Dr. Werner.*—What has struck me most forcibly in the paper is the part pertaining to the treatment of pyorrhœa. Now, do we understand that Dr. Smith uses the scalers and instruments first, and afterwards the treatment with the pumice and stick.

*Dr. Smith.*—Oh, certainly; get everything off. I tried to make it plain that I was even more thorough in the first treatment than Dr. Riggs, and in addition to that, to follow up the treatment perpetually. The difference is in the greater thoroughness and continuation of the treatment.

*Dr. Werner.*—And you do not treat systemically?

*Dr. Smith.*—You mean constitutionally? Not at all. I do not give them even the effervescent salts that are made down in Philadelphia especially for that purpose.

*Dr. Werner.*—You treat it wholly as a local expression or diseased condition? That is a very strong statement, and some may heartily agree with you on that. Another thing: do you put on the rubber dam in your method of treatment?

*Dr. Smith.*—No, never.

*Dr. Werner.*—You do not, as Dr. Hart, for instance, does, put on the rubber dam and sterilize?

*Dr. Smith.*—Not at all.

*Dr. Werner.*—Do you use iodine?

*Dr. Smith.*—Not at all. Water, pumice, and the stick, and muscle and friction.

*Dr. Werner.*—Well, you certainly must believe that a tooth-brush is a great adjunct and helper in massaging the gum and in removing the exudation around the necks of the teeth. You would not advise your patients not to use a tooth-brush?

*Dr. Smith.*—No. I advise them to use it thoroughly, and I scold them when they do not.

*Dr. Werner.*—And you would certainly say that a great deal of the success is due to their treatment?

*Dr. Smith.*—No, not much of it. If I could see them once in two weeks instead of once in four, I would not much care.

*Dr. Werner.*—Another strong statement the essayist made is that the odor, the fetid odor in the breath, is so very much due to the causes that he speaks of.

*Dr. Smith.*—Will you let me explain that right here?

*Dr. Werner.*—I believe that much more comes from the condition of the mucous membrane of the post-nasal region and from the deposit on the tongue than from any decayed condition of the teeth. I should think eighty per cent. of it came from abnormal conditions of those parts and very little from tooth decay.

*President Pond.*—We all remember a very interesting paper on this same subject that we listened to last year. It awakened a great deal of interest on account of the new application of old methods recommended and the wonderful results reported. You remember Dr. Taylor's mentioning, as I understood it, that he received his first impetus in the work from Dr. Smith. He spoke of cases that he was treating that he had had under his care for a limited time, perhaps six months or more. I would like to hear from him what the results are to the present time?

*Dr. Taylor.*—It is not my purpose this evening to discuss what Dr. Smith has said in regard to my paper. I had the pleasure of meeting you last spring; I received a very cordial greeting, and we will let the matter rest where it is. I wish to reinforce one statement that he has made this evening, the value to the human family by the frequent stimulation of tooth-substance by friction, the betterment of tooth-structure, I think Dr. Smith calls it, but it means one and the same thing. There is nothing done in dentistry to-day that is as valuable in the care of the mouth as the thorough stimulation of tooth-structure by friction. This is all I wish to say.

*Dr. Taft.*—I assume from Professor Smith's question to the essayist that the polishing-strip serves, in his opinion, a very useful purpose in the cleansing of teeth. I quite agree with him. The last patient that I had in my chair this afternoon, a little girl eleven years of age, will serve as an illustration to emphasize this point. With the exception of the upper and lower incisors, which were badly stained, her teeth were in what I would consider a perfect condition of cleanliness. After going over them with iodine and pumice, and with the help of the orange-wood stick, porte polishing-brushes, and dental engine, I noticed, as is generally the case, that the approximal surfaces could not be reached or freed from stain without the use of the separating saw and polishing-strip. This was done, and superficial decay discovered upon the distal surface of the right superior central. The polishing-strip in this instance served the double purpose of removing all stains and the prevention of further disintegration of tooth-tissue.

I understood the essayist to say, and correctly, I think, in answer to Professor Smith, that a thorough cleansing of the teeth by his method does not require their separation or the use of polishing-strips, and I refer to the case of which I have just spoken to emphasize the point that the orange-wood stick, no matter what its shape, or however pointed it may be, cannot be relied upon to do all that the essayist claims for it.

Disks of rubber or felt, the orange-wood stick, the separating saw, the polishing-strip, the porte polishing-brush, and the dental engine, each and all serve a useful purpose in any thorough performance of this work, and I should like to ask the essayist how, in a case like the one I have cited, for example, he would have removed all traces of stain, and how he would have discovered and arrested the process of decay without at least the use of the saw and the polishing-strip?

*Dr. Smith.*—I would have treated the tooth, I have no doubt, just exactly as the gentleman did. Credit me, gentlemen, with common sense. If I find decay on the approximal surface of the tooth, I remove it, filling if necessary. If I find a stain there, I take it off. If, as in this case, evidently, the teeth were only occasionally seen by this gentleman, it was found that decay had actually begun, what did he do? He did the common-sense thing, removed it, of course. Why not? Simply because you could not get your orange-wood stick in there. No, put your teeth in a perfect condition; that is the beginning of the treatment; and do it in the very best way it can be done.

*Dr. Taft.*—My point was, Mr. President, that without a separation of the incisors, no orange-wood stick alone, however pointed, could penetrate or remove the stain from the approximal surfaces of the incisors at their point of contact, much less be the means of discovering or of arresting superficial decay. The teeth had first to be separated, then sand-papered, and afterwards polished. The separation revealed caries, and was but an incident in the process of cleansing.

*Dr. Meriam.*—The paper seems to be rich in suggestions of possibilities, though much of the matter covers the ground that we have all covered, some in one way, and some in another. If it is an indication that dentistry is progressing past the sharp mechanical line into a physiological dentistry, then I think the paper is a forerunner of a most desirable change in our practice. In a recent paper regarding the practice in Germany, it was shown that even extraction of teeth was preceded by a thorough and intimate acquaintance with the anatomy of the jaw.

If we should have a practice of dentistry that is drawn from the conditions that we find, rather than based on appliances that we wish to use, we shall certainly have then a dentistry that is much advanced over the present time. You will remember a remark I made in the Academy some years ago, that the tendency of dentistry to-day is buying things in the shops and then trimming patients to fit them, and it is fair to presume that this is the kind of dentistry that the gentleman means when he refers to the gold work.

Too much tooth-substance has been cut away in order to make the use of certain instruments possible. I have seen fillings where the labial wall was cut away for the purpose of using a certain instrument. Now, if the paper goes beyond this, if it leads away from such practice, it is an advance. We have felt this practice less here because we are at a distance from shop influence, and have therefore less temptation to use entirely the ready-made.

One dealer said to me, "You do not let your patients dictate to you; why should you expect to dictate to us?" I said, "On the contrary, every patient dictates to us," and as we discover physiological and pathological conditions and bring the resources of dentistry to meet them, we gain reputation.

There are one or two practical points suggested. I think we had a discussion similar to this when Dr. Francis, of New York, read a paper. I spoke at that time of the use of the rattan stick in cleaning teeth. That can be shaved down on one side so it is as thin as a piece of steel, and holding the powder well. I think we owe the idea to Dr. J. L. Williams, of Boston. The "orange-wood" sold in our shops is not orange, but is, I believe, a kind of willow. It is sold in large quantities by dealers in watch-makers' tools, and is known as "peg-wood." It goes down the stairs of a watch-makers' supply house, then up another pair of stairs to a dentists' supply house, and changes to "orange-wood." It costs the watch-makers five cents a bunch, or fifty cents a dozen bunches.

*Dr. Smith.*—It does the work of cleaning teeth.

*Dr. E. H. Smith.*—Do I understand the essayist correctly when he says that he believes that all cases of pyorrhœa are produced by a mechanical irritation, and if this mechanical cause is removed and followed with a thorough cleansing of the teeth, the case necessarily goes on to a cure?

*Dr. D. D. Smith.*—Yes, that is practically my belief. I tried to state very clearly that I believe that this local irritant may often have a constitutional expression, but the constitutional expression never exists without previous local irritation, and consequently our treatment has always been, and must be, local rather than constitutional. Constitutional treatment alone for any case of pyorrhœa will be abortive. The common-sense treatment for it is what has been outlined, the changing of the environment of the teeth, keeping the surfaces perfectly clean, and giving nature a chance to do her restorative work.

*Dr. E. H. Smith.*—The hour is late, and I would not presume upon the patience of this audience to carry the discussion farther. I certainly feel the value of such a paper in so far as it treats of pyorrhœa, but I cannot, by my silence even, assent that all cases of pyorrhœa are produced by this means alone. I believe that there are many cases of pyorrhœa that have their preliminary steps in a constitutional trouble; for instance, in the case of a diabetic patient. I have had several such patients under my observation, and I find that when they are under treatment by physicians, and the diabetes is somewhat under control, there is a very great improvement in the condition of the gums. I believe there are many causes of pyorrhœa. For the pyorrhœa produced by mechanical irritation, from deposit of salts or exudations from the gum, as he states, his treatment is without question the proper treatment, and undoubtedly many of us have failed in not seeing our patients frequently enough and not carrying out this prophylactic treatment so thoroughly outlined.

*Dr. D. D. Smith.*—Allow me to say just one word in reply to that. He speaks of these cases of uræmic poisoning as being better when they are under constitutional treatment. I have to reply to that, that if he will put all such cases under the treatment which we have suggested, he will find that the constitutional expressions of uræmic poisoning will be much less decided. Do I make it clear?

*Dr. E. H. Smith.*—You make it clear, but at the same time I think that the treatment has got to be carried farther than that.

*Dr. D. D. Smith.*—We are not apt, as a profession, gentlemen, to agree upon the subject of pyorrhœa.

Subject passed.

### A UNIQUE METHOD OF TIGHTENING OR INSERTING LOWER FRONT TEETH.

BY DR. D. D. SMITH.

The evening is so far spent that I will not take a great deal of your time with this, but it is something that I have thought would interest you, possibly, more than the paper on prophylaxis. In all probability you will bring it more into requisition. And I rather desire, if it is your pleasure to accept it, that it may be presented to the profession from this society. I entitled it "A Unique Method of tightening or inserting Lower Front Teeth."

This original method of tightening loose lower front teeth, and of inserting artificial ones in vacant spaces, without plate or visible appliance, is dependent on a practice so heterodox when squared with the ordinary orthodox ideas and methods in dentistry that it has seemed more fitting to present it here rather in the form of a talk than as a set paper. It is my desire that it have free discussion of all means and methods advocated. A professor in one of our colleges remarked, on seeing the practical case which will be exhibited to you this evening, "That is all well enough in your hands, but I would not dare to teach it." To me the matter presents itself in this way: it is a legitimate and desirable method for these cases, or it is condemnatory and censurable. If the former, it ought to be known and taught; if the latter, it should be known to be avoided. This method has as its foundation the destruction of living pulps, usually in sound teeth, and hence diversity of opinion may arise respecting it.

A tooth without a vital pulp is not as good in some respects as one with a vital pulp. The pulpless tooth, in other words, if put to certain tests, as the tests of mechanical strain, is certainly not as strong as when its atoms are banded together by the vital force due to the presence of a living pulp.

But suppose we had some devitalized teeth in the mouth to be filled; what prognosis would we make in such event? There is scarcely a dentist in these days who would not make a favorable prognosis. We would say to the patient, we can save that tooth for you, in all probability, as long as you want it. Like any filled tooth, it may require refilling, it may be split or fractured, but practically the tooth is as good as if it had a living pulp.

I think it was put very clearly before the American Dental Association in 1899, in a paper on "The True Status of the Pulpless Tooth," that there are two sources of life to the tooth, the one an internal life, which is the nourisher of the tooth, the other an outside source of life, which unites it to the alveolus, and each entirely independent of the other. You can take away all the life from the internal parts of the tooth without in any way affecting the life distributed to the surface of the root of the tooth. It is the pericemental life of the tooth which we must maintain. We do not care so much about the pulp life; it is of very little account in certain conditions of the teeth. At my time of life, for instance, a vital pulp in a tooth is of little importance. We can preserve the tooth perhaps as well without as with it.

But how about the pericemental life? Destroy it, as in pyorrhœa, and you destroy the tooth. If we can preserve the pericemental life of the tooth in a perfect condition, we shall have a tooth for all practical uses equal to the tooth with the living pulp. The pulp is very important in young life, and we would by no means molest it then unless compelled to do so. Preservation of the tooth rests upon the treatment after devitalization.

Some men say, do not destroy a pulp in a tooth under any consideration. Why? Because they are afraid of the after-consequences.

You know how many methods there are for instantaneously destroying pulps. But can you destroy a pulp in a wisdom-tooth, for example, in that way? With perfect access to pulps, as in single-rooted teeth, no doubt the method is applicable. But there is a far better way, gentlemen, of destroying pulps, and that is with arsenical paste. Do you say an arsenical application will give pain? Not if it is properly applied. An arsenical application should never be made directly to a pulp, but always to living dentine. Complete devitalization will result in forty-eight hours, and there will be no pain attending it.

It is not necessary that the application should be deep in the tooth, but it must be in contact with living dentine. It must not be confined with cotton or any of the cements, but rather with some of the preparations of gutta-percha.

Now, another point. After devitalization, treatment for the removal of the pulp must be instituted, and the limit of time should be between forty-eight and sixty hours. I never use a nerve-broach for extracting a pulp. Why? Because I do not believe in them at all, and because they do not affect or bring about that condition of the root-canal which is requisite for its permanent comfort. We want to remove not only the pulp-tissue, but the membrane also which lines the canal, and we want, likewise, to remove a portion of the dentine from the root of the tooth; and this is the reason why it is best always to use the drill. The removal of the pulp with the drill insures free access to the root, and it takes from the root a portion of the dentine and of interdentinal matter which is subject to decomposition. It takes out that which always has been, and always will be, in cases of nerve-broach removal, a menace to the comfort of the tooth. I cannot now continue the details of pulp-canal treatment, but, assured that we are in the line of duty in devitalizing sound teeth, that they may be used as supports for sustaining other teeth, let us consider the little piece of work which I have to present to you this evening, and which I will now pass around for your inspection.

I want to say that I devised this method some eight years ago and have used it in many cases, both for tightening lower teeth and for the support of artificial ones, but it has taken me up to within the last six months to get it in its present perfected condition. This appliance is just as effectual for tightening loose teeth as for inserting artificial ones. If you have two tight teeth in which to place these supports, all will become immovable and secure. Where you have a case of pyorrhœa, for instance, and the alveolar process is nearly all absorbed away from the teeth, and where you want to retain the natural teeth and make them comfortable and useful, this appliance will enable you to do that. If any are gone, you can with the same appliance supply the missing ones. You will better understand how it is done by examining this little specimen than by any description which I can give it.

I have tied one of the little supports to the cast which slips into the right lower cuspid, and that shows the form of the support as first made and fitted into the tooth, and as this goes about I will endeavor to explain how to fit it. As you see the appliance you will divine how it is made and will understand the principle of the support. The cuspid teeth are almost always the ones to which you can attach this support. I have a practical case in the mouth of a patient here, who has been kind enough to come on with me, and I hope he is going to make a speech to you by and by, for he is *the* after-dinner speaker in Philadelphia. This cast is a representation of his mouth before the teeth were put in, and you will have an opportunity to see the case in its practical working.

The preparation of the teeth for the reception of this appliance is a very important part of the operation. I used to cut away from the cutting edge of the tooth, and from that down until I could get my supports in; but I no longer touch the cutting edge of the tooth, but go down near the gums to the thick portion of the tooth, where you can cut away without doing it any injury whatever. It took me five or six years, with the infrequent application of this principle, to discover the advantages of this more recent method of preparing the teeth. Cutting from the lingual face and into the thicker part of the crown, we can get ample space for strengthening the supports with solder without changing the form or enlarging the tooth.

Now, having cut from the palatine face as indicated, and enlarged the canal, take an eighteen-carat gold wire about 15

or 16 gauge and fit it into the root, giving it a sufficient length to get a real support. Next take a piece of No. 30 pure gold plate large enough to cover the lingual face of the tooth, make a hole through it, and push this pin or wire down into it; put the pin in its place in the tooth, carying the piece of gold plate with it, burnish the piece of gold onto the tooth itself at the point where it has been cut away. Cut off this piece of wire so the top of it will stand just above the tooth. Then you can make an impression, if you desire (I seldom do that), to enable you to handle it with ease until the final finishing of the case. Now just tack the piece of gold, after it has been fitted down on the tooth, with solder to the pin; after which you can adjust it perfectly to the tooth itself and contour the lingual attachment, strengthening it at the same time with solder. You want at least two of these supports in good firm teeth. There may be more of them if you like, as in this case, where I have three. The cuspid teeth are to be preferred for supports or piers.

Having the supports in the teeth as indicated, a plaster impression should be secured in which the supports will be securely embedded and may be removed without changing their relation to the teeth. Great care is necessary in making the cast from this impression, the teeth being small and usually long, but once made, the artificial teeth are to be fitted to it as though it were the mouth. You will notice that the gold on the lingual face in the specimen before you is shaped to perfectly represent the corresponding face of the natural teeth. The whole operation is a delicate one, and requires exact manipulation; it cannot be sent out to the mechanical dentist. You have to do the work yourself; at least, I have always had to do it. The teeth and the gold tubes are accurately fitted to the gums, a method which I use in all bridge-work. For facings I use the English (Ash & Sons) teeth almost exclusively.

With each individual tooth completed in its lingual construction and in its adjustment to the cast, they should be waxed together and to the supports in the plaster teeth. To avoid displacement after waxing, I cut away the plaster teeth in which the supports are embedded; this frees the piece from the cast when it is ready for investing and soldering, a part of the work which requires great care. When soldered and finished, the case is ready for adjustment.

One of the desirable features of this appliance is the fact that the rubber dam is always applicable in its adjustment. The dam should be applied to as many teeth as may be necessary to give untrammelled access to the teeth which carry the supports. Having applied the dam and completed the treatment of the roots, with a good cement the final adjustment of the appliance, whether it consists solely of these supports for the tightening of loose teeth, or whether it carries one or more teeth, is readily accomplished. Any length of time may be given the cements to harden, as all is completely protected by the dam.

The case, when completed, exhibits no gold, has the appearance of the natural teeth, is perfect in cleanliness, and presents nothing as unnatural or objectionable in the mouth to the patient.

It is difficult to describe in comprehensible terms details for the construction of an appliance such as this, but seeing this specimen, you will be fully able to catch its meaning. And now, if any one would like to ask questions before we examine the practical case which we have here, I shall be glad to answer them.

DISCUSSION.

*Dr. Meriam.*—How long have you had one of these in use?

*Dr. Smith.*—I made my first case, using this principle, fully ten years ago. I have had one in that mouth with pyorrhœa, that I was speaking of, for five years. It is not as good as this one is, because I had not then the process under control as now, but it is the same in principle.

I may be permitted to say that the gentleman who was in my office, apparently endeavoring to find fault with every condition possible, said of that case, which I regard as antiquated, "I cannot find any fault with that."

These cases are comparatively infrequent in practice, but the device is most satisfactory when it is needed. You will find many of these cases put in with bands around the teeth, exhibiting grotesque attempts at bridge-work. Frequently you will see loose teeth tied with gold strips or gilling twine.

*Dr. Brackett.*—May I ask Dr. Smith if he applies this principle sometimes in supporting an unbroken row of natural teeth where no artificial substitutes are needed?

*Dr. Smith.*—It was for this purpose that I first worked out the appliance. I prepare each loose tooth and fit into it a support after the manner described, and then solder all together, of course putting two of these posts into tight teeth.

*Dr. Brackett.*—In these cases do you ever grind away very much?

*Dr. Smith.*—Just the same as in cases with teeth. You have got to destroy the pulps in each tooth, hence there is no objection to cutting away from the lingual face of the tooth as you please to avoid any appearance or feeling of fulness.

*Dr. Brackett.*—In the advanced cases?

*Dr. Smith.*—Yes. Why not?

*Dr. Meriam.*—We notice at the bottom of this a white substance. What cement is it?

*Dr. Smith.*—Those tubes are filled with gutta-percha. You can fill them with anything you like, with vulcanite, if you wish, and vulcanize them in; but I have used the gutta-percha altogether for that purpose.

*Dr. Andrews.*—This is back of the teeth?

*Dr. Smith.*—Yes, in tubes which are formed to represent the lingual faces of the teeth. These tubes are filled with gutta-percha. They may be filled with cement or vulcanite and then vulcanized.

*Dr. Werner.*—These are Ash's teeth?

*Dr. Smith.*—The one standing alone is a White tooth, and the other two are Ash's; but I use Ash's teeth almost exclusively.

*Dr. Meriam.*—We understand you to say you use the Ash tube-teeth?

*Dr. Smith.*—No, the flat-tack, cross-pin teeth altogether.

*Dr. Piper.*—Why does the essayist have gutta-percha through here, so much surface in contact with the gum?

*Dr. Smith.*—That is the way I make all bridge-work. Made in this way it is much lighter, much stronger, less liable to accident, and, best of all, it can be kept in a good state of cleanliness.

On motion, adjourned.

CHARLES H. TAFT, D.M.D.,
*Editor American Academy of Dental Science.*

